# 177Lu-DOTA-0-Tyr3-octreotate infusion modeling for real-time detection and characterization of extravasation during PRRT

**DOI:** 10.1186/s40658-022-00466-y

**Published:** 2022-05-03

**Authors:** Christophe Mazzara, Julien Salvadori, Florian Ritzenthaler, Simon Martin, Clémence Porot, Alessio Imperiale

**Affiliations:** 1 Radiophysics, Institut de Cancérologie de Strasbourg Europe (ICANS), 17 rue Albert Calmette, 67093 Strasbourg, France; 2 Nuclear Medicine and Molecular Imaging, Institut de Cancérologie de Strasbourg Europe (ICANS), Strasbourg, France; 3 Radiopharmacy, Institut de Cancérologie de Strasbourg Europe (ICANS), Strasbourg, France; 4 Faculty of Medicine, FMTS, University of Strasbourg, Strasbourg, France; 5 Molecular Imaging – DRHIM, IPHC, UMR 7178, CNRS/University of Strasbourg, Strasbourg, France

**Keywords:** Extravasation, Peptide receptor radionuclide therapy, Gravity infusion method, Radioprotection, Equivalent dose rate, Neuroendocrine tumors, ^177^Lu, Lutathera

## Abstract

**Purpose:**

Given the recent and rapid development of peptide receptor radionuclide therapy (PRRT), increasing emphasis should be placed on the early identification and quantification of therapeutic radiopharmaceutical (thRPM) extravasation during intravenous administration. Herein, we provide an analytical model of ^177^Lu-DOTA0-Tyr3-octreotate (Lutathera^®^) infusion for real-time detection and characterization of thRPM extravasation.

**Methods:**

For 33 Lutathera^®^-based PRRT procedures using the gravity infusion method, equivalent dose rates (EDRs) were monitored at the patient’s arm. Models of flow dynamics for nonextravasated and extravasated infusions were elaborated and compared to experimental data through an equivalent dose rate calibration. Nonextravasated infusion was modeled by assuming constant volume dilution of ^177^Lu activity concentration in the vial and Poiseuille-like laminar flow through the tubing and patient vein. Extravasated infusions were modeled according to their onset times by considering elliptically shaped extravasation region with different aspect ratios.

**Results:**

Over the 33 procedures, the peak of the median EDR was reached 14 min after the start of the infusion with a value of 450 µSv h^−1^. On the basis of experimental measurements, 1 mSv h^−1^ was considered the empirical threshold for Lutathera^®^ extravasation requiring cessation of the infusion and start again with a new route of injection. According to our model, the concentration of extravascular activity was directly related to the time of extravasation onset and its duration, a finding inherent in the gravity infusion method. This result should be considered when planning therapeutic strategy in the case of RPM extravasation because the local absorbed dose for β-emitters is closely linked to activity concentration. For selected EDR values, charts of extravasated activity, volume, and activity concentration were computed for extravasation characterization.

**Conclusion:**

We proposed an analytical model of Lutathera^®^ infusion and extravasation (gravity method) based on EDR monitoring. This approach could be useful for the early detection of thRPM extravasation and for the real-time assessment of activity concentration and volume accumulation in the extravascular medium.

## Introduction

Given the recent rapid development of peptide receptor radionuclide therapy (PRRT), including ^177^Lu-DOTA-0-Tyr3-octreotate (Lutathera^®^) and ^177^Lu-PSMA-617 [1, 2], and the increasing number of patients previously treated with multiple lines of chemotherapy, increasing importance should be given to the prevention, detection and early quantification of therapeutic radiopharmaceutical (thRPM) extravasation during its intravenous administration.

Extravasation remains a rare phenomenon, but the consequences can nevertheless be serious due to localized tissue retention of thRPM and subsequent prolonged local exposure to ionizing radiation. To date, published data on the incidence and clinical outcome of radioactive extravasation are limited [3], and only a few cases summarize the effects of the extravasation of ^177^Lu-based PRRT during clinical procedures [4–8]. Specific guidelines have been published by the Society of Nuclear Medicine and Molecular Imaging (SNMMI), which provides practical advice in the case of RPM extravasation [9]. Given the physical characteristics of the radiation used (beta or alpha emitters) and depending on the injected volume and the administered activity, RPM extravasation can cause soft tissue damage peripheral to the injection site, ranging from simple skin desquamation [10] to radionecrosis [11–14].

In the case of early detection of thRPM extravasation, dispersive actions, such as warming, massaging and elevating the area of extravasation, are recommended to stimulate reabsorption of the radiopharmaceutical [5, 7]. However, surgery could also be considered, especially in the event of extravasation with a high concentration of activity. After extravasation, the success of therapeutic interventions is often assessed by repetitive measurements with probes or gamma cameras. Such measurements allow useful insight regarding the activity, volume and effective half-life of the extravasation and can ultimately lead to an estimation of the absorbed dose [5, 7, 13]. Preventive measures are also encouraged, such as the use of equivalent dose rate (EDR) monitoring during administration to prevent the accumulation of a large interstitial volume with consequent iatrogenic irradiation [15, 16]. In addition to allowing for early detection of extravasation, EDR monitoring when coupled with appropriate modeling of infusion flow dynamics could also be used for a real-time characterization of extravasation, which contribute to establish the appropriate therapeutic strategy.

In the present work, we propose an analytical model of Lutathera^®^ infusion (according to the gravity method) for early extravasation detection and real-time assessment of activity and volume accumulation in extravascular tissue.

## Materials and methods

This study was based on 33 procedures of Lutathera^®^-based PRRT performed in the setting of marketing authorization in 11 patients (6 women, 5 men, median age: 62 years) with metastatic grade I and II small-intestine neuroendocrine tumors progressing under cold somatostatin analogues treatment. The mean ± SD injected and residual activities, which were measured with a dose calibrator, were 7191 ± 140 MBq and 142.9 ± 121.3 MBq, respectively.

### Lutathera^®^ administration procedure

**Fig. 1:**
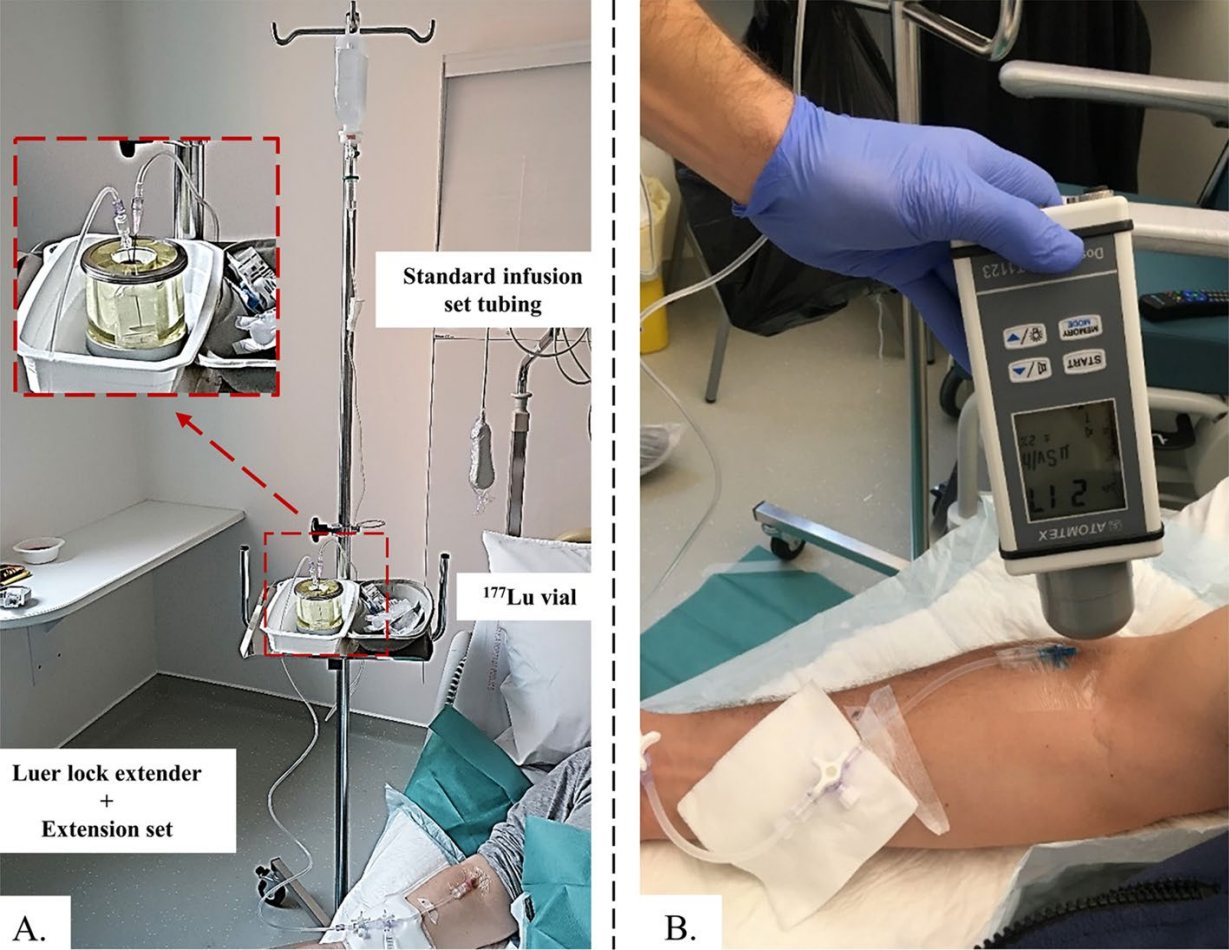
Illustration of the gravity infusion device (**A**) and equivalent dose rate measurements on the patient's arm (**B**)

thRPM was intravenously administered over approximately 40 min using the gravity infusion method [9, 17]. In summary, RPM administration was performed by directly infusing saline (NaCl 0.9%) into the vial with a gravity drip (Fig. 1A). The increased pressure in the vial pushes ^177^Lu-DOTATATE into the patient's intravenous line. The flow rate at the vial outlet is therefore imposed by the flow rate of the saline solution. In the first ten minutes, the flow rate was fixed at 0.25 drop s^−1^ (~ 50 mL h^−1^) followed by 0.5 drops s^−1^ from 10 to 20 min and finally at 1 drop s^−1^ from 20 min to the end of the infusion. Concomitant with the infusion of Lutathera^®^, an amino acid solution (LysaKare^®^ 25 g/25 g) was administered by contralateral intravenous infusion.

### Equivalent dose rate measurements

During each Lutathera^®^ infusion, the EDR was monitored by an expert operator with a AT1123 (Atomtex, Minsk, Republic of Belarus) survey meter at both the injection site (Fig. 1A) and the patient's abdomen. Measurements were performed every 2 min at a distance of 1 cm from the patient's skin (Fig. 1B). The AT1123 survey meter was calibrated in equivalent dose rate Hp(10) (µSv h^−1^) by APVL (France, Saint-Cyr-sur-Loire) with a beam of radiological quality (mean energy 83 keV) and an accuracy of ± 20%.

### Extravasation process modeling

#### Infusion model

**Fig. 2:**
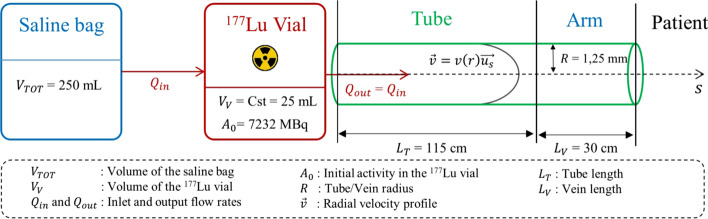
Simplified geometry used to model the infusion process

As illustrated in Fig. 2, a simplified model of the infusion process was proposed. The radius ($R$ = 2.5 mm) and length (${L}_{\mathrm{T}}$ = 115 cm) of the tubing line were modeled according to the manufacturer's specifications, and the vein in the forearm was considered to be the same diameter as the tubing line and 30 cm long. The long needle that joins the vial to the tube was not modeled. The flow rates entering and leaving the vial were considered equal at all times (${Q}_{\mathrm{out}}={Q}_{\mathrm{in}}=Q$), and the increase in flow rate at times ${T}_{1}$ = 10 min and ${T}_{2}$ = 20 min was taken into account. The influence of the blood pressure on the flow rate in the patient's arm was not modeled. The flow rates in the tube and the patient's arm were therefore assumed to be equal at all time. The total infusion time was fixed at ${T}_{\mathrm{inf}}$ = 40 min, and the radioactive decay was neglected as the ratio of the infusion time to the half-life of ^177^Lu was 0.004.

Assuming a homogeneous mixture, the following dilution equation for the activity in the vial (${A}_{V}$) can be considered:

1 $${A}_{V}(t)=\left\{\begin{array}{ll}{A}_{0}{.e}^{-{\alpha }_{1}.t} &\quad {\text{if}}\,\, t\le {T}_{1}\\ {A}_{0}.{e}^{-{\alpha }_{1}.{T}_{1}-{\alpha }_{2}.\left(t-{T}_{1}\right) }&\quad {\text{if}}\,\, {T}_{1}\le t\le {T}_{2}\\ {A}_{0}.{e}^{-{\alpha }_{1}.{T}_{1}-{\alpha }_{2}.\left({T}_{2}-{T}_{1}\right)-{\alpha }_{3}.\left(t-{T}_{2}\right)}&\quad {\text{if}}\,\, {T}_{2}\le t\le {T}_{\mathrm{inf}}\end{array}\right.$$

where ${A}_{0}$ is the median initial activity in the vial (7232 MBq) and $\left\{{\alpha }_{1}=\frac{{Q}_{1}}{{V}_{V}}, {\alpha }_{2}=\frac{{Q}_{2}}{{V}_{V}}, {\alpha }_{3}=\frac{{Q}_{3}}{{V}_{V}}\right\}$ are the dilution rate parameters imposed by the volume of vial (${V}_{V}$ = 25 mL) and the three consecutive flow rates (${Q}_{1}$ = 50 mL h^−1^, ${Q}_{2}$ = 100 mL h^−1^, ${Q}_{3}$ = 200 mL h^−1^).

In addition, the Reynolds number of the “tube + vein” compartment depends on the average flow velocity (${v}_{\mathrm{mean},j}, j=\left\{\mathrm{1,2},3\right\})$ and can be defined as follows:

2 $$\begin{aligned} {\text{Re}}_{j} & = \frac{{2.\rho .v_{{{\text{mean}},j}} .R}}{\eta } \\ v_{{{\text{mean}},j}} & = \frac{{Q_{j} }}{{\pi .R^{2} }} \\ \end{aligned}$$

Assuming a density and a dynamic viscosity of the diluent + thRPM mixture equal to that of saline solution (NaCl 0.9%) at room temperature (*ρ* = 1.0053 g cm^−3^, *η* = 1.02 × 10^−3^ Pa s [18]), the Reynolds number in the “tube + vein” compartment are 3.5, 7.0, 13.9 for the three consecutive flow rates. The flow regime is laminar of the Poiseuille type with a parabolic radial velocity profile (${v}_{j}\left(r\right), j=\left\{\mathrm{1,2},3\right\})$:

3 $${v}_{j}\left(r\right)={2.v}_{\mathrm{mean},j}\left(1-\frac{{r}^{2}}{{R}^{2}}\right)$$

**Fig. 3:**
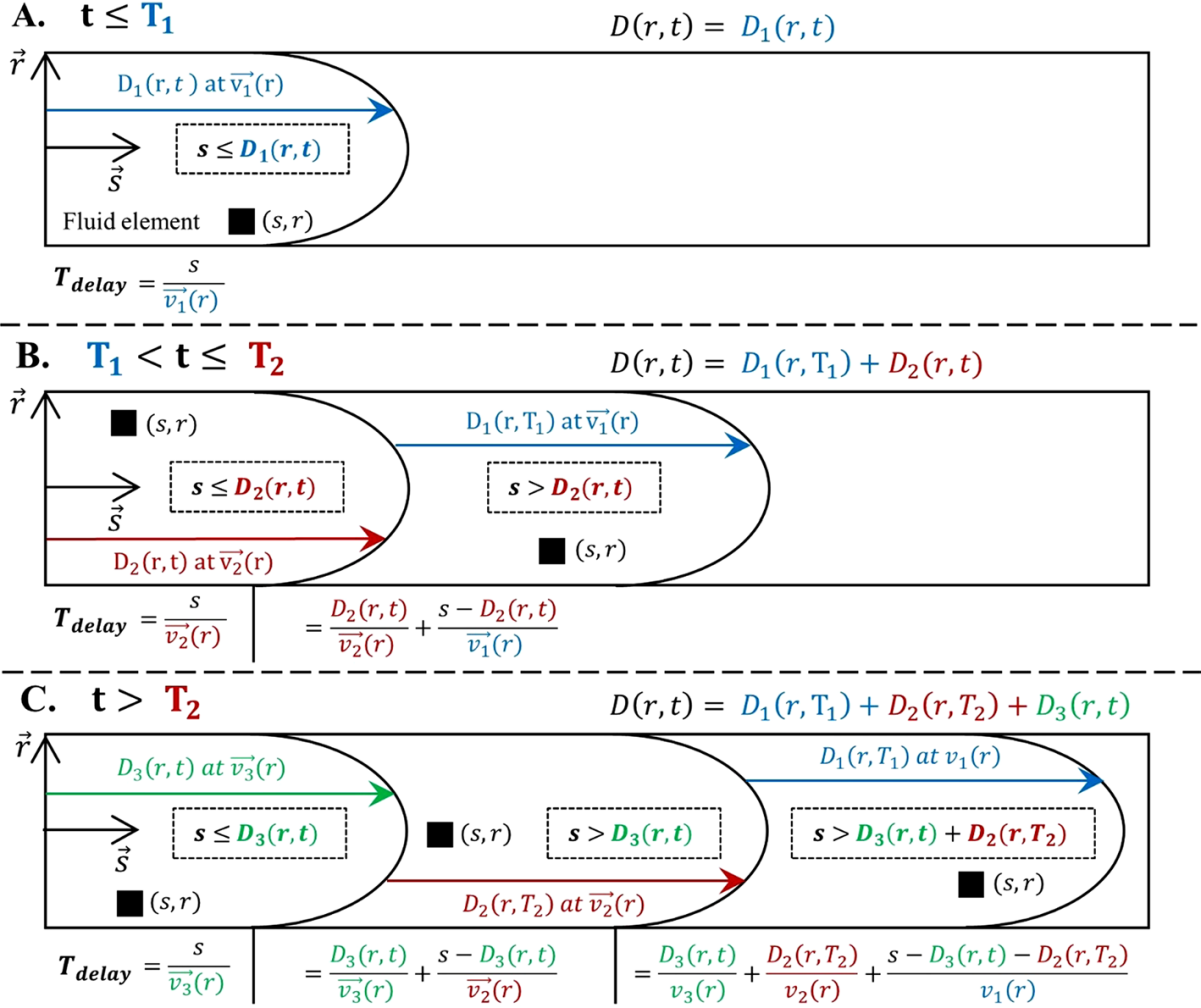
Illustration of the time delay (${T}_{\mathrm{delay}}$) between the vial concentration and the fluid element concentration as a function of time $t$ and the position of the fluid element in cylindrical coordinates $(s,r)$. **A** For $t < {T}_{1}$, the fluid element traveled the distance $s$ at the velocity $\overrightarrow{{v}_{1}}(r)$. ${T}_{\mathrm{delay}}$ is thus expressed as the ratio between the fluid element curvilinear abscissa $s$ and the velocity $\overrightarrow{{v}_{1}}(r)({T}_{\mathrm{delay}}=\frac{s}{\overrightarrow{{v}_{1}}\left(r\right) \, })$. **B** For ${T}_{1}<\mathrm{t }\le {T}_{2}$, there are two cases depending on the curvilinear abscissa s of the fluid element: Case 1. $s{\le D}_{2}(r,t)$, the fluid element has traveled the distance $s$ at the velocity $\overrightarrow{{v}_{2}}(r)\,({T}_{\mathrm{delay}}=\frac{s}{\overrightarrow{{v}_{2}}\left(r\right) \, })$. Case 2. $s>{D}_{2}(r,t)$, the fluid element has traveled the distance ${D}_{2}(r,t)$ at the velocity $\overrightarrow{{v}_{2}}(r)$ and the distance $s-{D}_{2}(r,t)$ at the velocity $\overrightarrow{{v}_{1}}(r)$ $({T}_{\mathrm{delay}}=\frac{{D}_{2}(r,t)}{\overrightarrow{{v}_{2}}(r) \, }+\frac{s-{D}_{2}(r,t)}{\overrightarrow{{v}_{1}}(r) \, })$. **C** For $t>{T}_{2}$, there are three cases depending on the curvilinear abscissa $s$ of the fluid element. The mathematical expression of ${T}_{\mathrm{delay}}$ is obtained by following the same method as for ${T}_{1}<t \le {T}_{2}$

Based on Eqs. (1), (2) and (3) activities in the tubing ($i=T$) and the arm ($i=A$) during the infusion can be expressed by the integral in cylindrical coordinates of the activity concentration (${C}_{V}=\frac{{A}_{V}}{{V}_{V}}$) in the corresponding curvilinear s-axis domains [${s}_{i}^{\mathrm{min}} {s}_{i}^{\mathrm{max}}$]:

4 $$\begin{aligned} & A_{i} \left( t \right) = \mathop \smallint \limits_{{s_{i}^{{{\text{min}}}} }}^{{{s_{i}{{\text{max}}}} }} \mathop \smallint \limits_{0}^{R} C_{V} \left( {t - T_{{{\text{delay}}}} \left( {s,r,t} \right)} \right).2\pi .r.{\text{d}}r.{\text{d}}s \\ & \begin{array}{*{20}c} {{\text{With}}\;\;C_{V} \left( {t - T_{{{\text{delay}}}} \left( {s,r,t} \right)} \right) = 0} & {{\text{if}}\;\;s > D\left( {r,t} \right)} \\ \end{array} \\ \end{aligned}$$

where $D(r,t)$ is the total distance traveled by the fluid at radius *r* and time *t* and ${T}_{\mathrm{delay}}(s,r,t)$ is the time for a fluid element to reach the coordinates (*s*, *r*) at time $t$. As illustrated in Fig. 3, these two parameters depend on the three consecutive radial velocity profiles (${v}_{j}\left(r\right), j=\left\{\mathrm{1,2},3\right\})$ according to the following equations:

5 $$D\left(r,t\right)=\left\{\begin{array}{ll}{{D}_{1}\left(r,t\right) =v}_{1}\left(r\right).t &\quad {\text{if}}\,\, t\le {T}_{1}\\ {{D}_{1}\left(r,{T}_{1}\right)+{D}_{2}\left(r,t\right) =v}_{1}\left(r\right).{T}_{1}+ {v}_{2}\left(r\right).\left(t-{T}_{1}\right)&\quad {\text{if}}\,\, {T}_{1}<t\le {T}_{2}\\ {D}_{1}\left(r,{T}_{1}\right)+{D}_{2}\left(r,{T}_{2}\right)+{D}_{3}\left(r,t\right)={v}_{1}\left(r\right).{T}_{1}+{v}_{2}\left(r\right).\left({T}_{2}-{T}_{1}\right)+{v}_{3}\left(r\right).\left(t-{T}_{2}\right)&\quad {\text{if}}\,\, {T}_{2}<t\le {T}_{\mathrm{inf}}\end{array}\right.$$

6 $${T}_{\mathrm{delay}}(s,r,t)=\left\{\begin{array}{ll}\frac{s}{{v}_{1}\left(r\right)} &\quad {\text{if}}\,\, t\le {T}_{1} \\ \frac{s}{{v}_{2}\left(r\right)} &\quad {\text{if}}\,\, {T}_{1}<t\le {T}_{2} \,\,{\text{and}}\,\, s\le {D}_{2}\left(r,t\right)\\ \frac{{D}_{2}\left(r,t\right)}{{v}_{2}\left(r\right)}+\frac{s-{D}_{2}\left(r,t\right)}{{v}_{1}\left(r\right)} &\quad {\text{if}}\,\, {T}_{1}<t\le {T}_{2} \,\,{\text{and}}\,\, s> {D}_{2}\left(r,t\right)\\ \frac{s}{{v}_{3}\left(r\right)} &\quad {\text{if}}\,\, t>{T}_{2} \,\,{\text{and}}\,\, s\le {D}_{3}\left(r,t\right)\\ \frac{{D}_{3}\left(r,t\right)}{{v}_{3}\left(r\right)}+\frac{s-{D}_{3}\left(r,t\right)}{{v}_{2}\left(r\right)}&\quad {\text{if}}\,\, t>{T}_{2} \,\,{\text{and}}\,\, {D}_{3}\left(r,t\right)+{D}_{2}\left(r,{T}_{2}\right)\ge s>{D}_{3}\left(r,t\right)\\ \frac{{D}_{3}\left(r,t\right)}{{v}_{3}\left(r\right)}+\frac{{D}_{2}\left(r,{T}_{2}\right)}{{v}_{2}\left(r\right)}+\frac{s-{D}_{3}\left(r,t\right)-{D}_{2}\left(r,{T}_{2}\right)}{{v}_{1}\left(r\right)} &\quad {\text{if}}\,\, t>{T}_{2}\,\,{\text{and}}\,\, s>{D}_{3}\left(r,t\right)+{D}_{2}\left(r,{T}_{2}\right)\\ \end{array}\right.$$

#### Extravasation model

Under normal conditions, the activity of the vial passes through the arm to reach the patient's body. The activity inside the tube at time $t$ is then provided by the infusion model (Eq. 4). In the event of extravasation, the activity is gradually stored in the arm and therefore no longer reaches the blood compartment. Assuming that extravasation does not affect the pressure in the “vial + tube” compartment, the dynamics of the vial and the flow rate in the tube remains unchanged. For extravasation occurring at injection site ${s}_{A}$ and time ${t}_{E}$, the extravasated activity in forearm (${A}_{E}(t,{t}_{E})$) at time $t\ge {t}_{E}$ can simply be expressed as the difference of activity in the “vial + tube” compartment between times ${t}_{E}$ and $t$:

7 $${A}_{E}\left(t,{t}_{E}\right)={A}_{V}\left({t}_{E}\right)+{A}_{T}\left({t}_{E}\right)-{A}_{V}\left(t\right)-{A}_{T}\left(t\right)$$

Depending on the values of the $(t,{t}_{E}$) couple with respect to the times ${T}_{1}$ and ${T}_{2}$, the extravasated volume in the arm at time $t\ge {t}_{E}$ is given by:

8 $${V}_{E}\left(t,{t}_{E}\right)=\left\{\begin{array}{ll}\left(t-{t}_{E}\right).{Q}_{1} &\quad {\text{if}}\,\, t\le {T}_{1} \\ \left(t-{t}_{E}\right).{Q}_{2} &\quad {\text{if}}\,\, {T}_{1}<t\le {T}_{2}\,\,{\text{and}}\,\, {t}_{E}>{T}_{1}\\ \left({T}_{1}-{t}_{E}\right).{Q}_{1}+\left(t-{T}_{1}\right){.Q}_{2} &\quad {\text{if}}\,\, {T}_{1}<t\le {T}_{2}\,\,{\text{and}}\,\, {t}_{E}\le {T}_{1}\\ \left(t-{t}_{E}\right).{Q}_{3} &\quad {\text{if}}\,\, {T}_{2}<t\le {T}_{\text{inf}}\,\,{\text{and}}\,\, {t}_{E}>{T}_{2}\\ \left({T}_{2}-{t}_{E}\right).{Q}_{2}+\left(t-{T}_{2}\right).{Q}_{3}& \quad {\text{if}}\,\, {T}_{2}<t\le {T}_{\text{inf}} \,\,{\text{and}}\,\, {t}_{E}\le {T}_{2}\\ \left({T}_{1}-{t}_{E}\right).{Q}_{1}+\left({T}_{2}-{T}_{1}\right).{Q}_{2}+\left(t-{T}_{2}\right).{Q}_{3}& \quad {\text{if}}\,\, {T}_{2}<t\le {T}_{\text{inf}}\,\,{\text{and}}\,\, {t}_{E} \le {T}_{1}\end{array}\right.$$

The concentration of extravasated activity can then be expressed as follows:

9 $${C}_{E}\left(t,{t}_{E}\right)=\frac{{A}_{E}\left(t,{t}_{E}\right)}{{V}_{E}\left(t,{t}_{E}\right)}$$

### Conversion to equivalent dose rates

#### Survey meter calibration

**Fig. 4:**
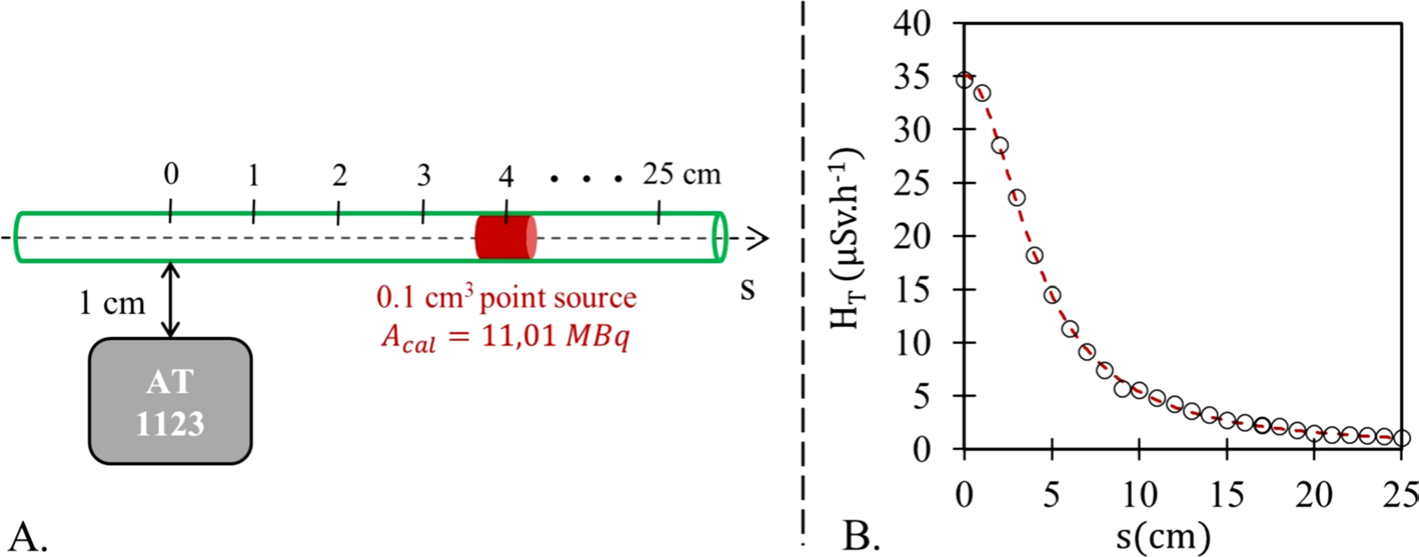
**A** Illustration of the calibration procedure of the AT1123 survey meter according to the position of an 11.01 MBq point source along the axis of the tubing(s) and for a fixed tubing-survey meter distance of 1 cm. **B** Result of the calibration that provides the relationship between EDR measurements and positions of the point source (circular symbols) with the corresponding regression (dashed line)

A proper calibration procedure of the AT1123 survey meter theoretically allows equivalent dose rates (EDRs) to be estimated from the simulated activities. This calibration was performed using a ^177^Lu calibrated point source (${A}_{\mathrm{cal}}$ = 11.01 MBq) inserted in the same tube as the one used during the administration (i.e., with an internal diameter of 2.5 mm). The activity of the point source was determined using a dose calibrator by difference between the measurements of a syringe filled with Lutathera^®^ before and after injection of the point source into the tube. As illustrated in Fig. 4A, the AT1123 survey meter was then placed at a fixed distance of 1 cm from the tubing, and the EDR of the point source was measured according to its position along the $s$ axis. Twenty-five equidistant measurements were performed for source positions ranging from 0 to 25 cm. The following function was used to fit the relationship between EDR measurements ${(H}_{T})$ and positions of the point source $(s)$:

10 $${H}_{T}\left(s\right)=a.{\left({s}^{2}+{b}^{2}\right)}^\frac{c}{2}$$

where $a$, $b$, and $c$ are the coefficients providing the best fit (Fig. 4B). These coefficients and goodness-of-fit criteria (*R*^2^ and RMSE) are given in Table 1.

**Table 1:** Parameters of the function used to model the relationship between equivalent dose rates measurements ${(H}_{T})$ and positions of the point source $(s)$

Regression parameters			Goodness of fit	
${H}_{T}\left(s\right)=a.{\sqrt{{s}^{2}+{b}^{2}}}^{c}$			${R}^{2}$	$\mathrm{RMSE }\,({\upmu\,{\mathrm {Sv}} }\,{\mathrm{h}}^{-1})$
$a\,({\upmu {\mathrm{Sv}} }\,{\mathrm{h}}^{-1}\, {\mathrm{cm}}^{-c})$	$b$ (cm)	$c$		
516.4	4.065	− 1.917	0.9994	0.267

Two goodness-of-fit criteria are additionally provided for each regression: the coefficients of determination (${R}^{2}$) and the root mean square error (RMSE)

#### Infusion model

EDR during the infusion may be estimated at 1 cm from the injection site ($${s}_{a})$$ by integrating the product of the theoretical activity concentration by the function $${H}_{T}$$ over a domain ranging from $$s={s}_{a}-m$$ to $$s={s}_{a}+m$$:

11 $${\mathrm{EDR}}_{{s}_{a}}\left(t\right)=\frac{1}{{A}_{\mathrm{Cal}}}\underset{{s}_{a}-m}{\overset{{s}_{a}+m}{\int }}\left(\underset{0}{\overset{R}{\int }}{C}_{V}\left({t-T}_{\mathrm{delay}}(s,r,t)\right).2\pi .r.\mathrm{d}r\right).{H}_{T}\left({s}_{a}-s\right)\mathrm{d}s$$

*m* is set at 20 cm because only activity present within ± 20 cm of the injection site is considered to contribute to the EDR measurements.

#### Extravasation model

Although the EDR during nonextravasated infusion could be estimated by a linear integration of weighted activity (Eq. 11), the EDR during extravasation requires integration over a spatial extent. In our study, we considered the extravasation regions to be elliptical in shape, centered on the injection site ($${s}_{a})$$ and homogeneous in activity concentration.

In addition, the thickness of the extravasation region is fixed to the diameter of the tubing (2.R) to be in line with the conversion function ${H}_{T}\left(r\right)$. Under these conditions, EDR may be estimated 1 cm above the ${s}_{a}$ point by integrating the function ${H}_{T}$ over an elliptical disc:

12 $${\dot{D}}_{{s}_{a}}\left(t\right)=\frac{{C}_{E}(t,{t}_{E})}{{A}_{\mathrm{Cal}}}\underset{0}{\overset{2\pi }{\int }}\underset{0}{\overset{r(\theta )}{\int }}{H}_{T}\left(r\right).r.\mathrm{d}r.\mathrm{d}\theta$$

where *r*(*θ*) is the polar equation of an ellipse whose semi major axis *β* and semi minor axis *α* are parallel and perpendicular to the arm direction, respectively:

13 $$r\left(\theta \right)=\frac{\beta }{\sqrt{1-{\mathrm{cos}}^{2}\left(\theta \right)+{\left(\frac{\beta }{\alpha }\right)}^{2}.{\mathrm{cos}}^{2}\left(\theta \right)}}$$

For a given extravasation region, the elliptical aspect ratio ($\mathrm{AR}=\frac{\beta }{\alpha }$) is fixed, and the $\beta .\alpha$ product is conditioned by the extravasated volume (${V}_{E}$) according to the following equation:

14 $$\beta .\alpha = \frac{{V}_{E}(t,{t}_{E})}{2R\pi }$$

### Implementation

The tubing and vein were sampled with 1 mm bins along the curvilinear abscissa $s$ and 0.005 mm along the radial direction $r$. The infusion model (Eq. 4) and the corresponding EDR (Eq. 11) were computed for times ($t$) ranging from 0 to ${T}_{\mathrm{inf}}$ = 40 min with a 1-min sampling. Elliptical extravasation regions were sampled with 0.5° bins along the angular direction $\theta$ and 0.1 mm along the radial direction $r$. The extravasation model (Eqs. 7, 8 and 9) and the corresponding EDR (Eqs. 12, 13 and 14) were computed for both times ($t$) and extravasation times (${t}_{E}$) ranging from 0 to ${T}_{\mathrm{inf}}$ with a sampling time of 1 min. As EDR measurements are strongly dependent on the shape of the elliptical extravasation region, EDRs were simulated for four different extravasation aspect ratios ($\mathrm{AR}$ = 2, 3, 4, and 5).

These calculations were performed using Python 3.6. All the processes, including the extravasation model and the conversion from MBq into µSv h^−1^, take approximately 1 h on a single core i7 8700 K @ 3.7 GHz processor.

## Results

### Equivalent dose rate measurements

For the 33 infusions included in this study, the fluid level in the vial was constant over the duration of the infusion and no Lutathera^®^ extravasation was observed on gamma camera images performed 6 h after the infusion for dosimetry purposes.

**Fig. 5:**
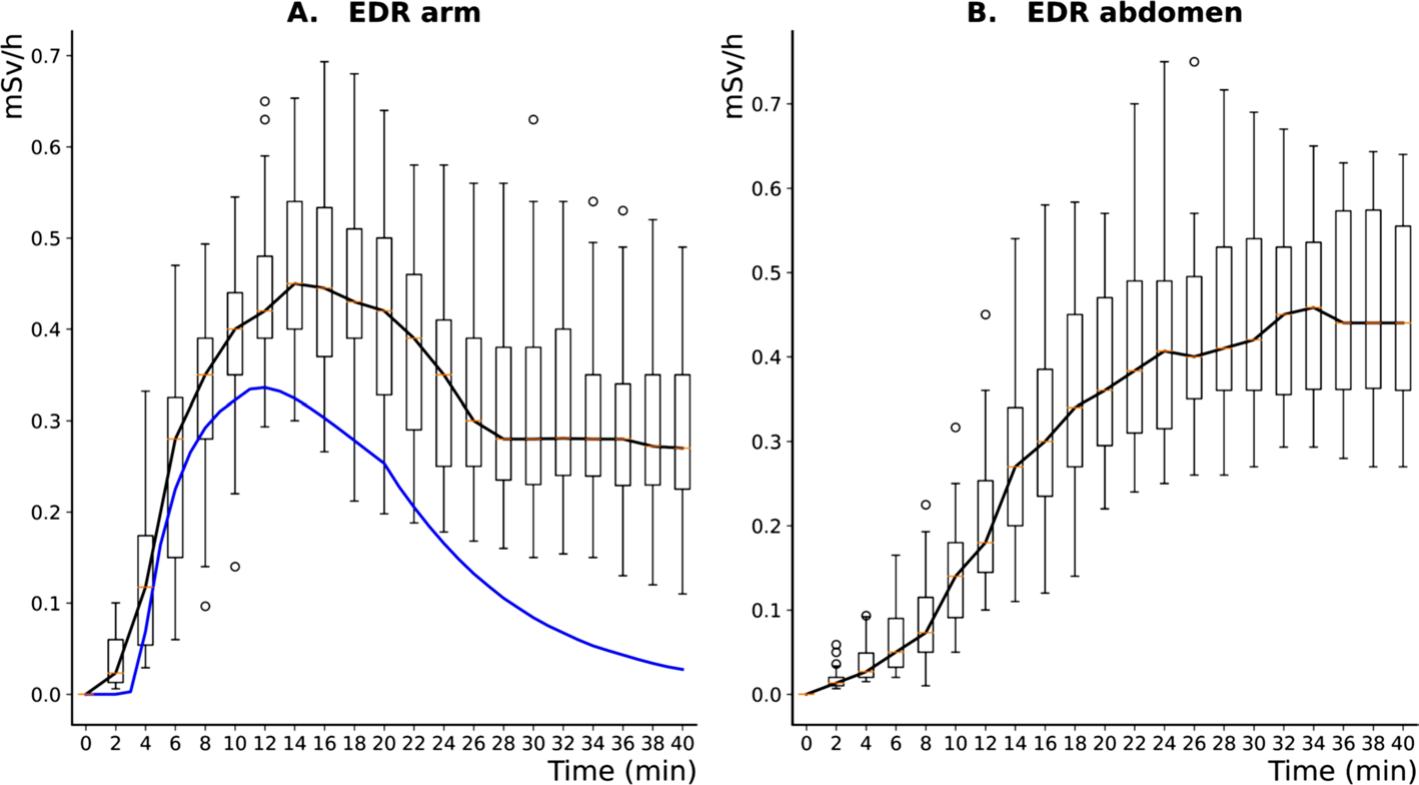
Box-plot representation of the evolution of experimental EDR at the patients’ arm (**A**) and abdomen (**B**) for the 33 Lutathera-based PRRT infusion procedures with the associated median value for each time point (black curve). The evolution of simulated EDR at the patient's arm with the proposed infusion modeling is also depicted (**A**, blue curve)

As evidenced in Fig. 5A, the EDR measured in the patient's arm (box plots) increased with the arrival of ^177^Lu and then peaked before decreasing. The peak of the median EDR over the 33 procedures (black curve) was 450 µSv h^−1^ and was reached 14 min after the start of the infusion. Accordingly, 1 mSv h^−1^ was considered an empirical threshold suggesting Lutathera^®^ extravasation, thus requiring the end of the infusion.

As depicted in Fig. 5B, the EDR measured in the patient's abdomen increased progressively with ^177^Lu infusion until reaching a plateau 20 to 25 min after the start of the PRRT procedure.

A high dispersion of intra- and interpatient measurements was observed due to the variability of the experimental conditions (see “Discussion” section).

### Extravasation process modeling

#### Nonextravasated infusion model

**Fig. 6:**
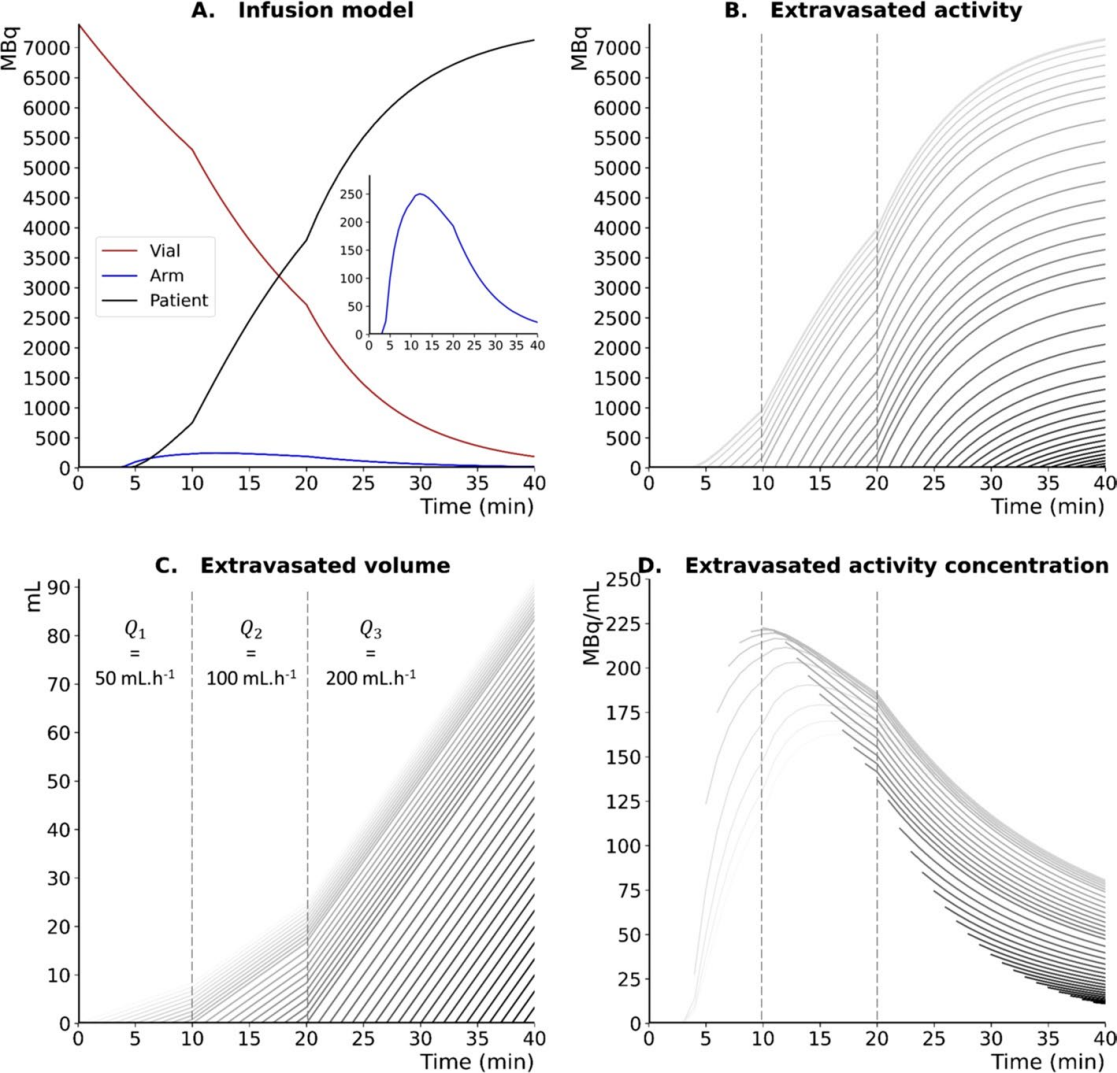
Evolution of simulated activity in the vial (red curve), arm (blue curve) and abdomen (black curve) for nonextravasated infusion (**A**). Evolution at the patient’s arm of simulated activity (**B**), volume (**C**) and activity concentration (**D**) for infusions that extravasate with onset times ranging from 1 to 40 min with 1-min sampling (gray curves)

The dynamics of the simulated activities in the vial (red curve), arm (blue curve) and patient (black curve) for nonextravasated infusion using the gravity method are represented in Fig. 6A.

According to our model, the activity of the vial decreases exponentially with the dilution of the ^177^Lu solution to reach a residual mean activity of 184 MBq at the end of the infusion (${T}_{\mathrm{inf}}$ = 40 min), and this finding is consistent with experimental measures from 33 PRRT procedures (143 MBq) with a relative difference of 29%. After a transit time in the tubing of approximately 3 min, the simulated activity in the arm increases relatively slowly (due to the parabolic radial velocity profile of the Poisseuille flow) to reach its maximum value 12 min after the start of the infusion. Then, the simulated activity decreases with the decay of the activity concentration imposed by the vial dilution.

In the patient, the activity follows the inverse dynamics of the vial with a delay of approximately 5 min corresponding to the transit time in the tubing and the vein.

#### Simulated versus experimental EDR for nonextravasated infusion

As depicted in Fig. 5A, good agreement was found between the dynamics of the simulated EDR (blue curve) and of the median experimental EDR (black curve) with a relative difference in time-to-peak of 14% (12 vs. 14 min, respectively). However, the proposed modeling underestimates the measured values, especially at the end of the infusion, due to the unmodeled contribution of the activity within the patient to the experimental EDR measurements (see “Discussion” section).

The significance of this contamination can be appreciated by analyzing the EDR measurements on the patients' abdomen (Fig. 5B). Values increase markedly until stabilizing 25–30 min after the beginning of perfusion.

#### Extravasated infusion model

**Fig. 7:**
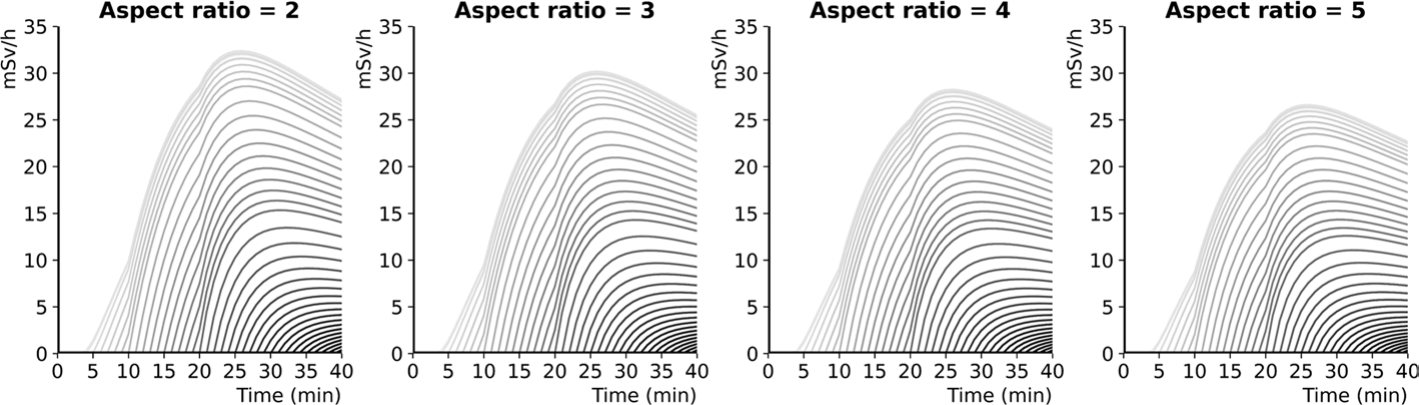
Evolution of simulated EDR at the patient’s arm for infusions that extravasate with onset times ranging from 1 to 40 min with 1-min sampling (gray curves) and for four elliptical aspect ratios (2, 3, 4 and 5)

The temporal evolution of activity, volume and activity concentration for simulated thRPM infusions according to the onset of extravasation is graphically depicted in Fig. 6B, C, D, respectively. Accordingly, rapid increases in both extravasated activity (Fig. 6B) and volume (Fig. 6C) were observed in the patient's arm. For a given flow rate, the extravasated volume increases linearly with the duration of extravasation. This is not the case for the extravasated activity concentration that ultimately decreases regardless of when extravasation begins mainly due to the exponential decrease of activity concentration in the vial (Fig. 6D). For early extravasation (${t}_{E}$ < 12 min), the extravascular concentration first increases before decreasing. The rate of increase of the extravascular activity concentration is related to the properties of the Poisseuille flow. In addition, this increase is slower but lasts longer for extravasations that start before the radiopharmaceutical reaches the arm (${t}_{E}$ < 3 min) due to the additional extravasated dilution volume. The simulated EDR at the patient’s arm during extravasated infusion is graphically presented in Fig. 7 for four elliptical aspect ratios. Regardless of the time of extravasation, the EDR value increases with the accumulation of the activity and decreases following vial dilution over time. The EDR depends on the ellipse aspect ratio: the higher the ellipse aspect ratio is, the lower the EDR at the patient's arm.

#### Extravasated volume, activity and concentration charts

**Fig. 8:**
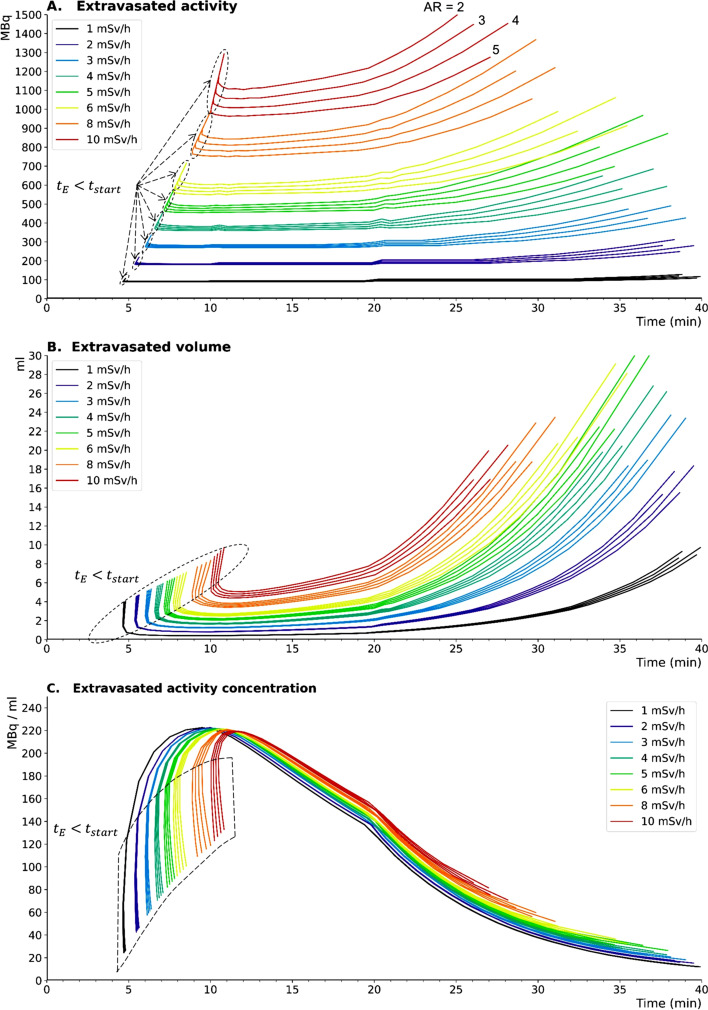
Range of extravasated activity (**A**), volume (**B**), and activity concentration (**C**) versus time for 4 aspect ratios (AR = 2, 3, 4, 5) that leads to the same EDR and for 8 representative EDR values (1, 2, 3, 4, 5, 6, 8 and 10 mSv/h)

Based on the extravasation modeling (Fig. 6B, C, D) and the corresponding EDR assessed for 4 different elliptical aspect ratios (Fig. 7), abacuses for volume, activity and activity concentration were computed for various values of EDR (1, 2, 3, 4, 5, 6, 8 and 10 mSv h^−1^, Fig. 8). These abacuses allow direct estimation of the volume, activity, and activity concentration at the patient's arm when extravasation occurs during PRRT infusions monitored by EDR measurements. The thickness of each iso-EDR domains (i.e., the range of extravasated activity as a function of time resulting in the same EDR) is related to the selected aspect ratios. For a given EDR, each aspect ratio value yields 1D curves of extravasated volume, activity and activity concentration versus time (Fig. 8).

Since it is difficult to experimentally quantify the spatial extent of an extravasation, all AR values were considered, which are represented in Fig. 8 by a given color. In compliance with the precautionary principle in radiation protection, the value corresponding to the highest EDR (i.e., the one corresponding to an aspect ratio of 2) should be used. Consequently, characterization of extravasation requires only two parameters: the EDR measured at the patient's arm and the time of the measurement relative to the start of the infusion. In practice, 20 min after the start of the injection, the measurement of an EDR of 3 mSv h^−1^ allows us to estimate an extravasated activity of 300 MBq, a volume of 2.2 mL and a concentration of 136 MBq mL^−1^. The shape of the first portion of EDR curves is related to the early extravasations (${t}_{E}$ $<$ ${t}_{\mathrm{start}}$) during which the extravasated volume is first filled with saline before the activity in transit through the tubing reaches the arm at time ${t}_{\mathrm{start}}$. At this point, the extravasation already has a volume of several cm^3^. Since the EDR is strongly correlated to the spatial extent (i.e., the dose rate decreases with increasing extravasated volume), a higher activity (in a higher volume) is needed for extravasation starting at ${t}_{E}$ $<$ ${t}_{\mathrm{start}}$ than at ${t}_{E}$ $=$ ${t}_{\mathrm{start}}$ to obtain the same EDR.

## Discussion

In the present study, we provided an analytical model of Lutathera^®^ infusion using the gravity method. Intravascular thRPM infusion was modeled by assuming constant volume dilution of ^177^Lu activity concentration in the vial and Poiseuille-like laminar flow through the tubing and patient vein. Extravasated thRPM infusions were modeled according to their onset times by considering elliptically shaped extravasation with different aspect ratios.

The gravity method involves constant volume dilution of the vial activity (Fig. 6A, red curve). During normal infusion (i.e.: without extravasation), the activity concentration at the patient's arm increases slowly due to the parabolic velocity profile of the Poiseuille flow and then decreases with vial dilution (Fig. 5A, blue curve). In the case of extravasation, the extravascular volume increases linearly over time with a slope given by the flow rate (Fig. 6C), whereas the activity concentration decreases as a result of the dilution of the vial (Fig. 6D). Thus, depending on the beginning and duration of extravasation, the activity concentration will be different. This result should be considered when planning the therapeutic strategy in the case of RPM extravasation because the local absorbed dose for β-emitters is closely associated with the activity concentration [19]. A highly concentrated small volume extravasation could be initially asymptomatic and induces a high absorbed dose with a higher risk of developing tissular necrosis (deterministic effects) than a more abundant and therefore more dilute thRPM extravasation [3]. This observation led us to calibrate our model to establish a relationship between the simulated extravasated activity, volume, and activity concentration and an easily measurable parameter, such as the EDR, at the patient's arm. Using this relationship, specific abacuses were built to estimate the physical characteristics of thRPM extravasation starting from a simple EDR measurement. To the best of our knowledge, this is the first time that extravasation kinetics modeling was coupled with an EDR calibration for in vivo real-time assessment of the activity concentration and thRPM volume accumulation in the extravascular medium. However, our analytical approach was based on a series of assumptions with consequent uncertainties and limitations. First, the influence of blood pressure and the variation of this pressure in case of extravasation was not modeled. The flow rate of the Poiseuille flow was therefore the same in the infusion tube and in the patient's arm, even in case of extravasation. Second, extravasation region was assumed to be elliptical in shape with a fixed thickness of 2.5 mm, and only 4 elliptical aspect ratios were considered. Third, extravasation was assumed to be homogeneous in activity concentration. Given these limitations, it should be noted that the proposed method has not been designed for dosimetric purposes. Within the framework of patient radiation protection, it is an additional tool for the clinician to detect and characterize the severity of extravasation in real time and to quickly determine the most relevant treatment option. Although the overall simulated results of this study were conditioned by the characteristics of the thRPM infusion (i.e., injection time and infusion rates), they may likely be reproduced adapting all infusion parameters according to the specific administration protocol used.

Based on EDR monitoring at the patient’s arm for 33 Lutathera-based PRRT infusion procedures without extravasation, 1 mSv h^−1^ was considered the empirical threshold for Lutathera^®^ extravasation requiring stopping the infusion. A certain degree of dispersion of intra- and inter patient EDR measurements was observed (Fig. 5) and mainly explained by the variability of the distance between the detector and the patient arm during EDR monitoring, the variable patient’s arm position, and the variable patient body mass index (self-attenuation). The use of a survey meter with a remote probe connected directly to the injection site (patient’s arm) could be useful to reduce operator-related measurement variability [15].

To evaluate the accuracy of the proposed modeling, simulated EDRs for nonextravasated infusions were compared with experimental data. Good agreement was found between the kinetics of the experimental and simulated EDRs with a relative difference of 14% between the simulated and experimental (median value) time-to-peak (12 vs. 14 min). However, the proposed modeling underestimates the measured values, especially at the end of the infusion, due to the unmodeled contribution of the activity within the patient to the experimental EDR measures. Nevertheless, this contribution could be measured on the patient's contralateral arm and subtracted from the EDR measured near the injection site to reduce measurement inaccuracy.

A recent paper by Tylski et al. presents the case of a large extravasated volume following Lutathera^®^ infusion with the gravity method [8]. In this case, the residence time in the arm was low (*T*_eff_ < 3 h) due to a rapid lymphatic drainage and the estimated doses (between 2 and 7 Gy) were in the lower range of deterministic effects and far under soft tissue necrosis threshold. However, considering the extravasation properties described in the present study for the infusion gravity method, such a large extravasated volume corresponds (from a dosimetric point of view) to the most favorable case. As stated above, the activity concentration of an extravasation with the gravity infusion method is not constant over the infusion; it decreases with the dilution of the vial and depends on both the onset and duration of this adverse event. The extravasation of a large volume is therefore strongly diluted, which mitigates the absorbed dose. It is also likely that a large extravasated volume is more rapidly drained by the lymphatic system than a small subcutaneous extravasation. Thus, a surgical intervention might be discussed to avoid any risk of necrosis in cases of early, low-volume, highly concentrated extravasation, which constitutes the most unfavorable case. In addition, the gravity method can have potential issues related to vial pressure and leakage. For these reasons, an infusion pump could be a safe alternative for Lutathera^®^ administration [20, 21]. With the pump, the concentration of the administered activity was constant over time. Consequently, in the case of extravasation, the activity concentration of the extravascular volume is independent to the beginning and duration of extravascular thRPM accumulation, limiting necrosis risks. A head-to-head comparison of Lutathera^®^ infusion methods (gravity vs. pump) is ongoing in our institution.

## Conclusion

Herein, we proposed an analytical model of Lutathera^®^-based PRRT procedures using the gravity method. This approach allows characterization of extravasated activity, volume, and activity concentration considering both the onset and the duration of thRPM extravascular accumulation. This method could be used as an additional radiation protection tool during PRRT procedures for early detection and real-time in vivo characterization of extravasation.

## Data Availability

The datasets used and/or analyzed during the current study are available from the corresponding author on reasonable request.
